# Dental management of patients taking novel oral anticoagulants (NOAs): Dabigatran

**DOI:** 10.4317/jced.53219

**Published:** 2017-02-01

**Authors:** Adrian Curto, Alberto Albaladejo, Alfonso Alvarado

**Affiliations:** 1 Department of Surgery, Faculty of Medicine, University of Salamanca, Salamanca, Spain

## Abstract

**Background:**

A new group of oral anticoagulants (dabigatran, rivaroxaban, apixaban and edoxaban) with clear advantages over classic dicoumarin oral anticoagulants (warfarin and acenocoumarol) has been developed in recent years. Patients being treated with oral anticoagulants are at higher risk for bleeding when undergoing dental treatments.

**Material and Methods:**

A literature search was conducted through April 2016 for publications in the ISI Web of Knowledge, PubMed and Cochrane Library using the keywords “dabigatran”, “rivaroxaban”, “apixaban”, “edoxaban”, “new oral anticoagulants”, “novel oral anticoagulants”, “bleeding” and “dental treatment”.

**Results:**

There is no need for regular coagulation monitoring of patients on dabigatran therapy. Whether or not to temporarily discontinue dabigatran must be assessed according to the bleeding risk involved in the dental procedure to be performed.

**Conclusions:**

The number of patients under treatment with new oral anticoagulants will increase in the coming years. It is essential to know about the pharmacokinetics and pharmacodynamics of new oral anticoagulants and about their interactions with other drugs. It is necessary to develop clinical guidelines for the perioperative and postoperative management of these new oral anticoagulants in oral surgical procedures, and to carefully evaluate the bleeding risk of dental treatment, as well as the thrombotic risk of suppressing the new oral anticoagulant.

** Key words:**Dabigatran, rivaroxaban, apixaban, edoxaban, novel oral anticoagulants, bleeding.

## Introduction

The number of patients under treatment with anticoagulant and antiplatelet drugs that require dental treatment has grown in recent years, so that there are many anticoagulated patients in our area. Oral anticoagulants and antiplatelet drugs are used for primary and secondary prevention of venous thromboembolic diseases (1).

Vitamin K antagonist anticoagulant drugs have been widely used for the treatment of nonvalvular atrial fibrillation. Atrial fibrillation is the first cause of embolism, regardless of whether it is associated to valve disease or not. For decades, it has been standardly treated with dicoumarin anticoagulants, mainly acenocoumarol and warfarin (2). Their therapeutic window is narrow and patients require regular International Normalized Ratio (INR) monitoring. Vitamin K antagonist drugs show many interactions with other drugs and with certain food.

The ideal oral anticoagulant should be safer and more effective than classic oral anticoagulants. In recent years, research in the area of pharmacology has focused on the development of new oral anticoagulants with a wide therapeutic window, few drug-drug and food-drug interactions, low intraindividual variability and no need for regular and systematic monitoring.

The four new oral anticoagulants that have been developed and are currently marketed are dabigatran (Pradaxa®), rivaroxaban (Xarelto®), apixaban (Eliquis®) and edoxaban (Lixiana®). Rivaroxaban, apixaban and edoxaban are direct Factor Xa inhibitors, while dabigatran is a direct thrombin inhibitor (3).

The efficacy of dabigatran in the treatment of nonvalvular atrial fibrillation was assessed in 2009 in the RE-LY study, which compared the use of warfarin versus dabigatran using a sample of 18113 patients with nonvalvular atrial fibrillation. The study showed a reduction in cardiovascular events in patients treated with dabigatran as compared to those taking warfarin (4,5).

According to the European Medicines Agency (EMA), the dose of dabigatran for the prevention of stroke and systemic embolism in patients with nonvalvular atrial fibrillation is between 110 and 150 mg taken orally every 12 hours (6).

Dabigatran exerts its anticoagulant effect through plasmatic binding to thrombin, thus deactivating it. It is a potent, selective, reversible and competitive thrombin inhibitor, and its possibility to reach the clot and deactivate the thrombin is one of the advantages of these new oral anticoagulants.

By inhibiting thrombin, dabigatran reduces the formation of fibrin, inhibits thrombin-mediated platelet activation and reduces fibrinolysis inhibition (7).

After oral administration of dabigatran etexilate, it is quickly metabolized to its active form by esterases in the intestine. Plasma concentrations peak within 2 hours of its administration and decrease by half after 12 hours (8).

 Table 1 shows the pharmacological characteristics of new oral anticoagulants (9).

**Table 1:**
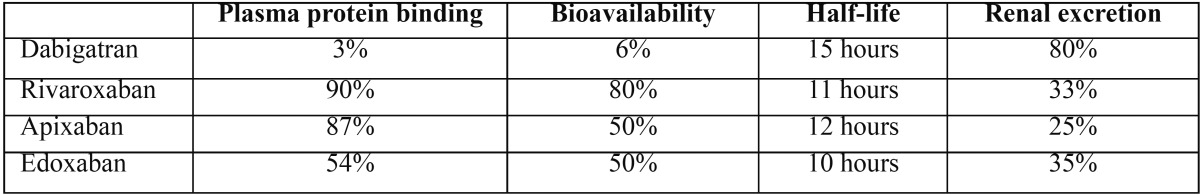
Pharmacological characteristics of new oral anticoagulants.

A shortcoming that was noticed during the initial stages of the development of these new drugs was the absence of antidotes to reverse their anticoagulant effect in comparison with classic Vitamin K antagonist anticoagulant drugs. In recent years, substances able to reverse the effect of new oral anticoagulants have been developed, among them idarucizumab (specific reversal agent of the anticoagulant effect of dabigatran), adexanet alfa and ciraparantag. In October 2015, the Food and Drug Administration (FDA) approved the marketing of idarucizumab (Praxbind®) as an antidote to the anticoagulant effect of dabigatran. Idarucizumab rapidly and completely reversed (within minutes) the anticoagulant activity of dabigatran in 88 to 98 % of patients in an initial clinical study. The approval of idarucizumab will substantially influence the approach to haemorrhagic complications related to dabigatran. It is worth noting that there is currently scarce clinical experience in the use of these new substances (10).

In economic terms, the cost of treatment with dabigatran is higher than with warfarin or acenocoumarol (11).

## Material and Methods

The aim of this paper is to contribute to the discussion on how to approach patients taking dabigatran, before, during and after dental treatment in light of the more recent knowledges.

In the present contribution we offer an exhaustive review of the literature found in the ISI Web of Knowledge, PubMed and Cochrane Library in April 2016, including articles published in the last 10 years. The words used were “dabigatran”, “rivaroxaban”, “apixaban”, “edoxaban”, “new oral anticoagulants”, “novel oral anticoagulants”, “bleeding” and “dental treatment” with the “and” boolean operator.

## Results

-Analytical tests:

Regular routine monitoring of the anticoagulant effect of new oral anticoagulants is not required, although it is necessary to be aware of their anticoagulant effect in the event of bleeding before starting invasive treatments. The effects of dabigatran can be quantified through different coagulation tests. New oral anticoagulants affect classic analytical tests in a dose-dependent manner. This entails a change of perspective in laboratory tests related to the perioperative management of new oral anticoagulants with regard to classic oral anticoagulants (12).

The ecarin clotting time, thrombin time and diluted thrombin time are sensitive to dabigatran plasma concentrations. Prothrombin time and activated partial thromboplastin time show low sensitivity to dabigatran. The determination of anti-Xa concentrations is the most specific test to measure dabigatran plasma concentrations (13-16).

-Dental management:

Patients under treatment with oral anticoagulants are at higher risk for bleeding when undergoing invasive dental treatments (17).

It may be necessary to temporarily discontinue dabigatran depending on the risk for bleeding involved in the dental treatment to be carried out and the patient’s renal function. Patients being treated with oral anticoagulants who have impaired renal function are at higher risk for bleeding (the fact that approximately 80% of dabigatran is excreted via the kidneys should be taken into account) (18,19).

Several studies have proved that the risk for bleeding of patients under treatment with new oral anticoagulants is similar to that of patients being treated with warfarin when International Normalized Ratio (INR) values are between 2 and 3 (20,21).

Based on the risk for bleeding involved, dental treatments can be classified into procedures with a low bleeding risk and procedures with medium or high bleeding risk.

-Perioperative management in dental procedures with low bleeding risk:

Temporary discontinuation of dabigatran is not necessary prior to invasive dental procedures with low bleeding risk, which include simple exodontia, oral surgery of up to 45 minutes and periodontal surgery with minimal bleeding risk (22-25). Hemostasis is to be facilitated, with local measures designed to help healing and minimize the risk of bleeding.

-Perioperative management in dental procedures with medium and high bleeding risk:

These cases (multiple extractions > 3, surgery lasting more than 45 minutes and head cancer surgery) require discontinuation of dabigatran and the need to consider subcutaneous heparin as an alternative treatment. There has been controversy for years regarding the suspension or alternation of anticoagulant therapy when planning invasive dental treatments. The instructions to discontinue dabigatran should be consulted with the specialist physician (26-29).  Table 2 provides guidance for discontinuation of dabigatran.

**Table 2:**
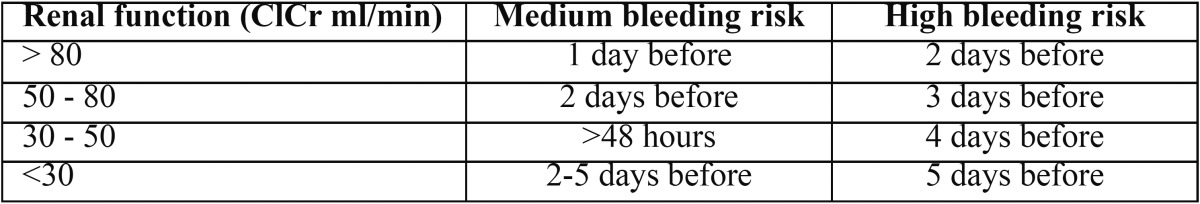
Instructions to discontinue dabigatran in dental procedures with medium and high bleeding risk.

-Postoperative management in dental procedures:

Management must be optimized from preoperative workup to the postoperative period in all patients subjected to oral anticoagulant treatment.

When to resume dabigatran depends on the extent to which the patient’s haemostasis is satisfactory. Treatment with dabigatran should be restarted as soon as possible. In dental procedures with average or high bleeding risk, dabigatran can be restarted 24-48 hours after surgery (30,31).

Local haemostatic measures such as sutures, gelatine or cellulose sponges, and tranexamic acid mouthwashes are required to help reduce the possibility of postoperative bleeding. Good soft tissue management is essential in this sense, in order to avoid excessive traumatism of the surgical zone (32).

The management of haemorrhagic complications in patients under treatment with dabigatran should be individualized according to the severity of the bleeding, the first step being to temporarily discontinue dabigatran.

In cases of mild bleeding it is enough to stop the oral anticoagulant and use local haemostatic measures. In patients with renal insufficiency, the half-life of dabigatran can increase, which may worsen bleeding (33,34).

In moderate to serious bleeding it is essential to treat the patient in a hospital. Haemodialysis treatment, administration of a transfusion of platelet concentrates, parenteral administration of antifibrinolytics or prothrombin complex concentrates or recombinant factor VIIa is essential in cases of moderate to severe bleeding (35).

-Drug interactions:

Although dabigatran is not metabolized by the cytochrome P450 enzymes, it is a substrate of P-glycoprotein. This relationship defines the main interactions of dabigatran with other drugs.

While dabigatran does not interact directly with nonsteroidal anti-inflammatory drugs (NSAIDs), these should be prescribed with caution. It is preferable to administer analgesics (36).

Pharmacodynamic interactions with other antithrombotic and antiplatelet agents have not been fully studied yet. The combined use of acetylsalicylic acid and dabigatran should be avoided, since it increases the risk of bleeding (37).

Proton pump inhibitors (omeprazole, esomeprazole, lansoprazole, rabeprazole, pantoprazole) slightly reduce the intestinal absorption of dabigatran (38).

The use of erythromycin and clarithromycin should be avoided because of their interaction with the effect of dabigatran (39). This is also the case with rifampicin, which reduces dabigatran plasma concentrations (40).

## Conclusions

The number of patients under treatment with new oral anticoagulants around us is increasing.

Dabigatran is a valid alternative to vitamin K antagonists for oral anticoagulation in patients with nonvalvular atrial fibrillation.

Health professionals should be aware of the pharmacokinetics and pharmacodynamics of new oral anticoagulants, and it is necessary to establish clinical guidelines for the perioperative and postoperative management of dental patients being treated with these new drugs.
